# Three Decades of Contact Lens-associated Microbial Keratitis in a Referral Hospital in São Paulo, Brazil

**DOI:** 10.5935/0004-2749.20210079

**Published:** 2021

**Authors:** Jorge Agi, Talita Trevizani Rocchetti, Maria Cecília Zorat Yu, Michelle Lima Farah, Fabio Ramos, Denise de Freitas, Ana Luisa Höfling-Lima

**Affiliations:** 1 Department of Ophthalmology and Visual Sciences, Escola Paulista de Medicina, Hospital São Paulo, Universidade Federal de São Paulo, São Paulo, SP, Brazil

**Keywords:** Contact lenses/adverse effects, Eye infections, bacterial/microbiology, Acanthamoeba keratitis, Corneal ulcer, Lentes de contato/efeitos adversos, Infecções oculares bacterianas/microbiologia, Ceratite por *Acanthamoeba*, Úl cera de córnea

## Abstract

**Purpose:**

The aim of this study was to analyze patient data and the laboratory results of corneal samples collected from patients followed at the Ophthalmology Department, Hospital São Paulo, Brazil over a 30-year period, and correlate the analize with contact lens wearing.

**Methods:**

Corneal samples from patients diagnosed clinically with microbial keratitis between January 1987 and December 2016 were included in this study. Cultures that were positive for bacteria, fungi, and *Acanthamoeba* spp. were analyzed retrospectively. To ascertain if the number of patients with contact-lens-associated microbial keratitis (as a risk factor for microbial infection) changed over time, the analysis was divided into three decades: 1987-1996, 1997-2006, and 2007-2016. Information pertaining to patient gender and age, as well as type of organism isolated, were compared among these periods.

**Results:**

The corneal samples of 10.562 patients with a clinical diagnosis of microbial keratitis were included in the study, among which 1.848 cases were related to the use of contact lenses. The results revealed that the frequency of contact-lens-associated microbial keratitis increased over the last two decades. Overall, females had contact-lens-associated microbial keratitis more frequently (59.5%). Patients aged 19-40 years also had contact-lens-associated microbial keratitis more frequently in all study periods. *Staphylococcus* spp. and *Pseudomonas* spp. were the most frequent Gram-positive and Gram-negative bacteria, respectively, in the microbial keratitis groups. Among the fungal cases of microbial keratitis, filamentous fungi were the most frequent fungi over the entire study period, with *Fusarium* spp. being the most frequent fungi in the group with microbial keratitis not associated with contact lens wearing (p<0.001). Samples positive for *Acanthamoeba* spp. and *Pseudomonas* spp. were significantly correlated with contact-lens-associated microbial keratitis (p<0.001).

**Conclusions:**

Females and young adults aged 19-40 years exhibited the highest frequency of contact-lens-associated microbial keratitis in our study. *Staphylococcus* spp. and *Fusarium* spp. were the predominant bacteria and fungi, respectively, isolated from corneal samples. *Pseudomonas* spp. and *Acanthamoeba* spp. were significantly correlated with contact-lens-associated microbial keratitis in this study.

## Abstract (pt)

**Objetivo:**

O objetivo deste estudo foi analisar da dos epidemiológicos de pacientes e resultados laboratoriais para todas as amostras de córnea coletadas de pacientes atendidos no Departamento de Oftalmologia do Hospital São Paulo, Brasil, durante um período de 30 anos e correlacionar com o uso de lentes de contato.

**Métodos:**

Amostras de córnea de pacientes com diagnóstico clínico de ceratite microbiana (de janeiro de 1987 a dezembro de 2016) foram incluídas neste estudo. Resultados laboratoriais para culturas positivas para bactérias, fungos e *Acanthamoeba* spp. foram analisados retrospectivamente. Para verificar se o número de pacientes com ceratite microbiana associada à lente de contato, fator de risco para infecção microbiana, mudou ao longo do tempo, a análise foi dividida em três décadas: 1987-1996, 1997-2006 e 2007-2016. As informações incluindo o sexo do paciente, idade e tipo de organismo isolado foram comparadas entre os períodos. A análise estatística foi realizada no software SAS/ STAT 9.3 e SPSS (v20.0).

**Resultados:**

Amostras de córnea de 10.562 pacientes com ceratite microbiana foram incluídas no estudo, das quais 1.848 foram relacionadas ao uso de lentes de contato. Os resultados revelaram que a frequência de ceratite microbiana associada à lente de contato aumentou nas últimas duas décadas analisadas. No geral, os homens compreendiam uma proporção maior do grupo ceratite microbiana não associada à lente de contato (CMNLC) (60,3%) e as mulheres eram mais frequentes no grupo ceratite microbiana associada à lente de contato (59,5%). Pacientes com idade entre 19 e 40 anos foram mais frequentemente observados no grupo ceratite microbiana associada à lente de contato em todos os períodos. *Staphylococcus* spp. foi a bactéria Gram-positiva mais frequentes, enquanto *Pseudomonas* spp. foi a bactéria Gram-negativa nos grupos ceratite microbiana. Entre os fungos ceratite microbiana, os fungos filamentosos foram os fungos mais frequentes durante todo o período do estudo, com *Fusarium* spp. sendo o mais frequente no grupo ceratite microbiana não associada à lente de contato. *Acanthamoeba* spp. e *Pseudomonas* spp. amostras positivas foram significativamente correlacionadas com ceratite microbiana associada à lente de contato.

**Conclusões:**

A maior prevalência de ceratite microbiana associada à lente de contato no nosso estudo foi observada em mulheres e adultos jovens com idade entre 19 e 40 anos. *Staphylococcus spp.* e *Fusarium spp.* foram as bactérias e fungos predominantes isolados nas amostras da córnea. *Pseudomonas* spp. e *Acanthamoeba* spp. foram significativamente correlacionados a ceratite microbiana associada à lente de contato neste estudo.

## INTRODUCTION

Microbial keratitis (MK) is an important cause of sight-threatening eye infection and is considered one of the leading causes of blindness in developing and developed countries^(1)^. In India, approximately 2 million people develop corneal ulcers every year^(2,3)^. In the United States, an estimated 30,000 cases of MK (including bacteria, fungi, and *Acanthamoeba* spp. related cases) are reported annually^(4)^. Brazil is a large country with a variable incidence of MK because of geographical differences. In the Southeastern region of the country, the percentage of MK among the total number of eye infections followed at the Ocular Microbiology Laboratory, Federal University of São Paulo (UNIFESP) is around 40%^(5)^.

Contact lens (CL) wearing, trauma, corneal surgery, ocular surface disease (e.g., tear deficiencies), systemic diseases, and immunosuppression are potential risk factors for MK^(6,7)^. With the increase in CL use, the impact of MK on eye health has become increasingly relevant. Risk factors for CL-associated microbial keratitis (CLMK) include overnight use of CLs, a higher number of days of use, male gender, younger age, inadequate cleaning of CLs, poor CL-storage and case-cleaning habits, and purchase via the internet^(8-11)^. Regarding the daily wear of CLs, given the associated benefits of an absence of requirement for the use of storage cases or lens-cleaning solutions, it was hoped that infectious complications would be avoided. Unfortunately, daily use of disposable CLs remains a risk factor for MK. Although environmental microorganisms represent the largest group of causative organisms for MK, such organisms are detected less frequently among wearers of daily disposable CLs^(12)^.

Typically, CLMK is related to bacterial infection^(13)^; however, *Acanthamoeba* and, less frequently, fungi can also be involved in the pathological process of this condition. Gram-negative bacilli represent the major bacterial agents in this context, with *Pseudomonas aeruginosa* being reported most frequently in previous studies^(8,10,14,15)^. The etiological agent that is usually responsible for MK varies according to geographical region^(16)^.

The aim of this study was to analyze the epidemiological and microbiological sample data collected from patients who were clinically diagnosed with MK over a 30-year period (sent to the Laboratory of Ocular Microbiology, Hospital São Paulo, Brazil) and to correlate them with CL use.

## METHODS

We retrospectively analyzed patient data and the laboratory results of corneal samples collected from patients who were followed at the Ophthalmology Department, Hospital São Paulo, Brazil. The analysis was divided into three 10-year periods (1987-1996, 19972006, and 2007-2016). The following data were examined: patient gender, age (classified as <18, 19-40, 41-60, and >61 years), CL wearing parameters, culture results (positive or negative), and etiological agents present in the samples (bacteria, fungi, and *Acanthamoeba* spp.).

Information about lens type, CL wearing modality, and CL hygiene was not evaluated in this study because of the difficulty of acquiring this information from clinical and laboratory medical records.

Scrapings were collected by a trained physician under slit-lamp visualization using a Kimura spatula and topical anesthesia. The material obtained from corneal scrapings was smeared onto two slides and stained with Giemsa and Gram stains. Corneal material was also added to thioglycolate (Thio) broth, brain heart infusion, and solid agar media (Sabouraud, blood, and chocolate). Additional slide stainingand/or culture media-related procedures were performed depending on the clinical suspicion. Non-nutrient agar with an overlay of *Escherichia coli* medium, for example, was always used when *Acanthamoeba* spp. infection was clinically suspected. All scrapings were performed according to the standard protocol of the Microbiology Ocular Laboratory, Hospital São Paulo. A positive result was determined according to Stapleton et al.^(12)^: “an organism was identified on more than one medium or on one solid medium with organism having the same morphology as the organism visualized in the corneal scrape by staining methods”. If the organism was recovered from only one medium and/or after long periods of incubation, the result was considered negative^(12)^.

Statistical analysis was performed via the Cochran-Armitage test to evaluate the existence of a trend in CLMK percentage among the three decades, using the SAS/STAT 9.3 software (SAS Institute Inc. 2011b); and by the chi-squares or Fisher’s exact test using the SPSS for Windows software (ver. 20.0; SPSS Inc., Chicago, IL, USA). A significance level of 5% was used for all statistical tests.

## RESULTS

We analyzed the corneal scrapings from 10.562 patients who received a clinical diagnosis of MK from January 1987 to December 2016. Among these patients with keratitis, 1.848 (17.5%) had CLMK. During the three periods analyzed, the incidence of MK and CLMK increa sed; however, the increase in CLMK was statistically significant from the first to the second decade, from 7.3% to 19.6% (p<0.001) (Table 1). The increase in MK positivity was accompanied by an increase in culture requests at our Medical Center.

**Table 1:** Number and percentage of NCLMK, CLMK, and MK cases in each period analyzed in the present study

	NCLMK		CLMK		Total (MK)	
	N	%	N	%	N	%
Period						
1987-1996	2148	92.7%	168	7.3%	2316	100.0%
1997-2006	3132	80.4%	762	19.6%	3894	100.0%
2007-2016	3434	78.9%	918	21.1%	4352	100.0%
Total	8714	82.5%	1848	17.5%	10562	100.0%

NCLMK= no contact lens-associated microbial keratitis; CLMK= contact lens-associated microbial keratitis; N= number.

Cochran-Armitage Trend Test (z=-12.1; *P*<0.001): 1987-1996 <1997-2006 = 2007-2016.

The positivity for bacteria and fungi in cultures increased in the MK and CLMK groups during the three decades analyzed. Among the MK positives samples that were positive for *Acanthamoeba* spp., 100% (13/13) of cases belonging to CLMK group in the first decade, 90% (197/218) in the second decade, and 87.6% (162/185) in the third decade (Table 2).

**Table 2:** Number of culture requests and positivity for bacteria, fungi, and *Acanthamoeba* in the CLMK group and total (MK) group during the period analyzed and during the three periods

	Period						Total		*P*
	1987-1996			1997-2006	2007-2016				
	N	%	N	%	N	%	N	%	
Total (MK)									
**Bacteria**	**2.183**	**100.0%**	**3.661**	**100.0%**	**4.167**	**100.0%**	**10.011**	**100.0%**	**<0.001**
Negative	1.475	67.6%	2.065	56.4%	1.342	32.2%	4.882	48.8%	
Positive	708	32.4%	1.596	43.6%	2.825	67.8%	5.129	51.2%	
**Fungi**	**2.081**	**100.0%**	**3.512**	**100.0%**	**3.981**	**100.0%**	**9.574**	**100.0%**	**<0.001**
Negative	1.961	94.2%	3.304	94.1%	3.647	91.6%	8.912	93.1%	
Positive	120	5.8%	208	5.9%	334	8.4%	662	6.9%	
***Acanthamoeba* spp.**	**36**	**100.0%**	**668**	**100.0%**	**1.483**	**100.0%**	**2.187**	**100.0%**	**<0.001**
Negative	23	63.9%	450	67.4%	1.298	87.5%	1.771	81.0%	
Positive	13	36.1%	218	32.6%	185	12.5%	416	19.0%	
**CLMK**									
**Bacteria**	159	**100.0%**	**676**	**100.0%**	**848**	**100.0%**	**1.683**	**100.0%**	**<0.001**
Negative	113	71.1%	375	55.5%	304	35.8%	792	47.1%	
Positive	46	28.9%	301	44.5%	544	64.2%	891	52.9%	
**Fungi**	**148**	**100.0%**	**642**	**100.0%**	**811**	**100.0%**	**1.601**	**100.0%**	**<0.001**
Negative	142	95.9%	629	98.0%	764	94.2%	1.535	95.9%	
Positive	6	4.1%	13	2.0%	47	5.8%	66	4.1%	
***Acanthamoeba* spp.**	**20**	**100.0%**	**439**	**100.0%**	**670**	**100.0%**	**1.129**	**100.0%**	**<0.001**
Negative	7	35.0%	242	55.1%	508	75.8%	757	67.1%	
Positive	13	65.0%	197	44.9%	162	24.2%	372	32.9%	

MK= microbial keratitis; CLMK= contact lens-associated microbial keratitis; N= number; *P*= Chi-squared test.

The incidence by gender differed between the MK and CLMK groups, as males were more frequent in the non-CL-associated MK (NCLMK) group (60.3%) and females were more frequent in the CLMK group (59.5%) (p<0.001). The incidence of CLMK increased in all four age groups between 1987 and 2016, with the incidence being highest among individuals aged 19-40 years for all three decades analyzed (55.2%, 64.1%, and 58.6%, respectively).

*Staphylococcus* spp. was the most frequent Gram-positive bacterium isolated from samples from both the NCLMK and CLMK groups. Among Gram-negative bacteria, *Pseudomonas* spp. was most frequently isolated from samples of both groups. Among *Staphylococcus* spp., *S. aureus* was the most frequently isolated agent from NCLMK (49.5%) and CLMK (45.7%) samples in the first period (1987-1996). However, in the second and third periods (1997-2006 and 2007-2016, respectively), coagulase-negative *Staphylococcus* (CoNS) was the most commonly isolated bacteria from the NCLMK group (36.2% and 51.6%, respectively) and the CLMK group (45.2% and 57.4%, respectively). *Streptococcus* spp. was more frequent in the NCLMK group compared with the CLMK group in the second and third periods (p<0.001). *Pseudomonas* spp. was more common in the CLMK group in the first and second periods (p<0.001) (Table 3).

**Table 3:** Prevalence of bacterial species in the NCLMK and CLMK groups in each period analyzed

	Period of 1987-1996				Period of 1997-2006						Period of 2006-2016				*P*
	NCLMK		CLMK		*P*	NCLMK		CLMK		*P*	NCLMK		CLMK		
	N	%	N	%		N	%	N	%		N	%	N	%	
CoNS	82	12.4%	4	8.7%	0.459	469	36.2%	136	45.2%	0.004	1.177	51.6%	312	57.4%	0.016
**S. *aureus***	328	49.5%	21	45.7%	0.609	171	13.2%	21	7.0%	0.003	280	12.3%	59	10.8%	0.356
***Streptococcus* spp.**	108	16.3%	4	8.7%	0.171	234	**18.1%**	25	8.3%	**<0.001**	269	**11.8%**	19	3.5%	**<0.001**
***Pseudomonas* spp.**	64	9.7%	17	37.0%	**<0.001**	142	11.0%	81	**26.9%**	**<0.001**	165	7.2%	64	11.8%	0.001
***Serratia spp.***	3	0.5%	2	4.3%	0.002	46	3.6%	19	6.3%	0.029	100	4.4%	39	7.2%	0.007
***Corynebacterium* spp.**	0	0.0%	0	0.0%	-	0	0.0%	0	0.0%	-	16	0.7%	1	0.2%	0.223^a^
**Other GPC**	6	0.9%	0	0.0%	1.000^a^	14	1.1%	6	2.0%	0.244^a^	72	3.2%	10	1.8%	0.001
**Other GPB**	0	0.0%	0	0.0%	-	62	4.8%	18	6.0%	0.393	321	14.1%	70	12.9%	0.456
**Other GNB**	80	12.1%	3	6.5%	0.257	147	11.4%	15	5.0%	0.001	148	6.5%	23	4.2%	0.047
**Others**	14	2.1%	1	2.2%	1.000^a^	47	3.6%	0	0.0%	0.001	18	0.8%	2	0.4%	0.400^a^
**GN Coccobacilli**	17	2.6%	0	0.0%	0.619^a^	104	8.0%	9	3.0%	0.002	120	5.3%	13	2.4%	0.004

NCLMK= no contact lens-associated microbial keratitis; CLMK= contact lens-associated microbial keratitis; *CoNS=* coagulase-negative *Staphylococcus; S. aureus= Staphylococcus aureus*; GPC= Gram-positive cocci; GPB= Gram-positive bacilli; GNB= Gram-negative bacilli; *P*= level of significance; N= number; GN Coccobacilli= Gram-negative Coccobacilli; *P*= Chi-squared test; (a) Fisher exact test.

Among all positive fungal cultures, filamentous fungi were the most frequent in all three periods for the MK group. *Fusarium* spp. was the most common filamentous species isolated from the NCLMK group for all three periods (>70%; p<0.001). *Candida albicans* was the most frequent yeast species isolated from the NCLMK group samples in the second period (p<0.001), which *C. parapsilosis* was the most frequent yeast species isolated in the third period for this group (p<0.001) (Table 4). In the CLMK group, *Fusarium* spp. was the most common filamentous species isolated for all three periods, while *C. albicans* was the most frequent yeast species in the second and third period. However, this difference was not statistically significant.

**Table 4:** Prevalence of fungal species in the NCLMK and CLMK groups in each period analyzed

	Period of 1987-1996					Period 1997-2006				Period of 2007-2016					
	NCLMK		CLMK		P^^1^^	NCLMK		CLMK		P^^1^^	NCLMK		CLMK		P^^1^^
	N	%	N	%		N	%	N	%		N	%	N	%	
Filamentous fungi	103	100.0%	4	100.0%	0.382	157	100.0%	6	100.0%	0.476	209	100.0%	25	100.0%	0.243
*Fusarium* spp.	56	**54.4%**	2	50.0%		104	**66.2%**	3	50.0%		114	**54.5%**	11	44.0%	
*Aspergillus* spp.	15	14.6%	0	0.0%		17	10.8%	1	16.7%		24	11.5%	2	8.0%	
*Penicillium* spp.	9	8.7%	1	25.0%		2	1.3%	0	0.0%		13	6.2%	1	4.0%	
*Paecilomyces* spp.	1	1.0%	0	0.0%		9	5.7%	0	0.0%		12	5.7%	5	20.0%	
Other hyaline fungi	6	5.8%	1	25.0%		9	5.7%	1	16.7%		12	5.7%	2	8.0%	
Dematiaceous fungi	4	3.9%	0	0.0%		13	8.3%	1	16.7%		34	16.3%	4	16.0%	
Not identified	12	11.7%	0	0.0%		3	1.9%	0	0.0%		0	0.0%	0	0.0%	
*P^^2^^*	<0.001			0.759			**<0.001**		0.572		<0.001			0.007	
**Yeast fungi**	11	**100.0%**	2	**100.0%**	0.039	38	**100.0%**	7	**100.0%**	0.178	78	**100.0%**	23	**100.0%**	0.268
C. *albicans*	10	90.9%	0	0.0		22	57.9%	5	71.4%		20	25.6%	8	34.8%	
*C. parapsilosis*	0	0.0%	1	50.0%		11	28.9%	0	0.0%		40	**51.3%**	7	30.4%	
*C. guilliermondii*	0	0.0%	0	0.0%		2	5.3%	0	0.0%		10	12.8%	3	13.0%	
Other *Candida* spp.	1	9.1%	1	50.0%		2	5.3%	2	28.6%		5	6.4%	4	17.4%	
Other yeast fungi	0	0.0%	0	0.0%		0	0.0%	0	0.0%		3	3.8%	1	4.3%	
Not identified	0	0.0%	0	0.0%		1	2.6%	0	0.0%		0	0.0%	0	0.0%	
*P^^2^^*		0.007		1.0			**<0.001**		0.257		<0.001			0.125	

NCLMK= no contact lens-associated microbial keratitis; CLMK= contact lens-associated microbial keratitis; *C. albicans= Candida albicans; C. parapsilosis= Candida parapsilosis; C. guilliermondii*= *Candida guilliermondii; P*= level of significance; N= number; *P*^^1^^= Fisher’s exact test for fungal distribution comparisons of keratitis type; *p*^^2^^= Chi-squared test for fungal type percentage in one sample.

Regarding co-infections, cultures showing more than one bacterial strain were the most frequent (53.3%). Cultures exhibiting more than one group of microorganisms (e.g., bacteria, fungi, and *Acanthamoeba* spp.) were also observed in the second and third periods. Co-infection by bacteria and *Acanthamoeba* spp. was more frequent (5.7%) than co-infection by bacteria and fungi (1.8%), and by bacteria, *Acanthamoeba* spp., and fungi (0.1%).

## DISCUSSION

CL wearing is one of the most important risk factors for MK^(7)^. Considering the large number of CL wearers worldwide and the fact that etiological profiles can vary according to geographical region^(17)^, epidemiological investigations are an important public health tool for the prevention of MK.

The consequences of daily CL wearing were the subject of numerous studies performed in the mid-1990s, in which CLs were shown to be a predisposing risk factor for MK. In the present study, we obtained results regarding the frequency of MK infection related to CL wearing over a 30-year period. Our results were similar to those of a previous study performed at the same healthcare center^(18)^, which showed that the frequency of CLMK increased in the past years. Retrospective and prospective studies have reported a relationship between CLMK frequency and CL type, i.e., soft (extended wear, daily disposable, and cosmetic) vs. rigid (corneal or scleral) lenses^(12,15)^. Despite the fact that the use of soft CLs remains the most important risk factor for the development of *Acanthamoeba* spp. keratitis, the recommendations for the use and care of scleral CLs should also be emphasized, as the use is not unrestricted from this type of infection^(19)^.

Several studies have reported that males are more commonly afflicted with CLMK than females^(20-22)^. In our study, females were predominant in the CLMK group, whereas males were more frequently found in the entire MK group. Lam (2002) also found a higher frequency of female vs. male CLMK cases^(23)^. It is well known that CL use provides a better appearance and imposes less restrictions on daily activities^(24)^. This may be the reason why our study showed a female predominance among CLMK cases. Most of the patients with CLMK in our study were young adults (19-40 years of age), corresponding to the age group described in other CLMK-related studies^(22)^.

Here, bacteria were the most frequent microorganisms isolated from the samples, followed by fungi and *Acanthamoeba* spp.. Compared with previous studies performed in other geographical areas, the microorganisms isolated from our samples were different^(13,16)^. In some countries, such as India and Nepal, fungal keratitis is the most frequent type of MK^(3,25)^. Conversely, bacteria are more common in patients with MK in developed countries^(10,16)^. Despite the fact that Brazil is a developing country, Gram-positive bacteria were always the most frequent microorganism isolated^(18)^ in our research center, even when different age ranges were analyzed^(26)^.

In the present study, *Acanthamoeba* spp. was the second-most frequent type of microorganism isolated from patients with CLMK; this result was similar to that of a previous study conducted in Hong Kong^(10)^. The high incidence of *Acanthamoeba* spp. detected among the CLMK samples in our study was also expected, as it had been described previously^(27)^. Here, the number of *Acanthamoeba* spp. positive samples was significantly higher in the CLMK group compared with the total MK sample. Over the 30-year period covered in this study, the requests for *Acanthamoeba* spp. culture at our healthcare center increased since the first reported case of MK caused by *Acanthamoeba* spp.^(28)^.

Gram-negative bacilli are commonly isolated from CLMK samples in tropical countries, whereas Gram-positive bacteria are more common in countries with a temperate climate^(10)^. *Staphylococcus* spp. was the most frequent species in the corneal samples of the NCLMK and CLMK groups in the current study. This was surprising because Brazil is a tropical country; thus, *Pseudomonas* spp. would be expected to be the most commonly isolated bacteria^(14-16)^. However, its location at the Tropic of Capricorn at a considerable elevation provides a rather subtropical-to-temperate climate to São Paulo. Conversely, *Pseudomonas* spp. was the major causative agent of bacterial CLMK in our experience (p<0.001).

The results of this study demonstrated that, among patients with MK, fungal infection was less common than bacterial infection; there was one fungal case for every eight bacterial cases. The probable explanation for this finding relies on the level of urbanization of São Paulo, as a higher percentage of fungal MK cases would be expected in rural environments^(29)^. *Fusarium* spp., *Aspergillus* spp., and *Candida* spp. are the most commonly isolated fungi worldwide in cases of corneal infection^(30,31)^. In the present study, we were able to demonstrate a higher frequency of filamentous fungal infection in the MK group from 1987-2016, with *Fusarium* spp. being the predominant fungal species.

In this study, co-infection by bacteria and *Acanthamoeba* spp. was more frequent than co-infection by: (i) bacteria and fungi; and (ii) bacteria, *Acanthamoeba* spp., and fungi. *Acanthamoeba* spp. keratitis polymicrobial infection is also observed as a secondary infection, with both bacteria and fungi^(32,33)^. Through endosymbiosis, *Acanthamoeba* spp. may inoculate bacteria, fungi, and viruses into the cornea, in which they cause keratitis^(34)^.

In conclusion, we described the epidemiological profile of MK over the past 30 years at our referral center in São Paulo, Brazil. During this period, CLMK was more frequent among females and young adults. Overall, in contrast to what it is observed in other tropical countries, Gram-positive bacteria (*Staphylococcus* spp.) were predominant among all corneal samples analyzed. Moreover, in accordance with other studies, *Fusarium* spp. was the most frequently isolated fungus. Finally, our study was able to significantly correlate CLMK to *Pseudomonas* spp. and *Acanthamoeba* spp. infections.
